# Isolated loss of inferior pubic ramus: a case report

**DOI:** 10.1186/1752-1947-2-202

**Published:** 2008-06-12

**Authors:** Aly Saber

**Affiliations:** 1 Port Fouad General Hospital, Port Fouad, Port Said, Egypt

## Abstract

**Introduction:**

It has been stated that regulation of the development of the iliac bone is different from that of the ischium and pubis. There are well-known clinical syndromes concerned with hypoplasia of ischiopubic bone, such as small patella syndrome, nail-patella syndrome, ischiopubic-patellar hypoplasia, and ischiopubic hypoplasia.

**Case presentation:**

A fit and otherwise healthy 35-year-old woman presented with pain in the left lower limb of 6 months duration. She sought advice from an orthopedic surgeon and was referred for exclusion of a primary soft tissue neoplasm. There was no history of trauma, chronic medical illness or surgical operations. Full systemic examination, laboratory investigations and whole body imaging showed no soft tissue swelling or any other bony defects. Isolated loss of the left inferior pubic ramus and thinning of the superior pubic ramus were detected, raising the question of whether the lesion was a secondary osteolytic lesion, a primary osteolytic lesion or due to endocrine disease.

**Conclusion:**

Isolated loss of the inferior pubic ramus with no concomitant bony or soft tissue anomalies is previously unreported. To the best of the author's knowledge, this finding has not been described previously.

## Introduction

The development of the pelvic girdles has been poorly investigated and reported evidence suggests that the regulation of ilium development is different from the development of ischium and pubis [1].

An extensive study was carried out to investigate the prenatal development and mineralization of ossification centers in the pelvic bone (ilium, ischium, and pubic bone) using radiography and optical density measurements on human fetuses. The mineral density of the pelvic bone increases with age and the mineralization rate changes throughout fetal life [2]. The hip bone is ossified from eight centers: three primary, one each for the ilium, ischium, and pubis; and five secondary, one each for the crest of the ilium, the anterior inferior spine, the tuberosity of the ischium, the pubic symphysis, and one or more for the Y-shaped piece at the bottom of the acetabulum. At birth, the three primary centers are quite separate and by the seventh or eighth year, the inferior rami of the pubis and ischium are almost completely united by bone [3].

Delayed ossification of limbs and girdles is an expression of several congenital syndromes and dysplasias, and major morphological abnormalities can arise at any time during the fetal period, with deformation more frequent in the third trimester when the fetus is subjected to greater constraint [4]. There are well-known clinical syndromes associated with hypoplasia of ischiopubic bone, such as small patella syndrome, nail-patella syndrome, ischiopubic-patellar hypoplasia, and ischiopubic hypoplasia [5].

## Case presentation

A fit and otherwise healthy 35-year-old woman presented with pain in the left lower limb of 6 months duration. She had sought advice from an orthopedic surgeon and was referred for exclusion of a primary soft tissue neoplasm. She complained of pain in the left hip joint that worsened with walking and long periods of standing; there was no complaint regarding either knee. She denied any history of trauma, violence or abnormal muscular overload. There was no history of chronic medical illness or surgical operations. There was no family history of congenital defects or similar conditions.

On examination she was apparently well-built, fit and otherwise healthy, and full systemic examination with special attention to both breasts and thyroid showed no soft tissue swelling or any other bony defects. There was no evidence of any congenital anomalies especially in the genitalia, hip bones or the long bones of the lower limbs or chest. Laboratory investigations were performed and showed normal organ functioning. Serum calcium and phosphorus and a parathyroid hormone assay were carried out. All were within the normal ranges.

**Figure 1:**
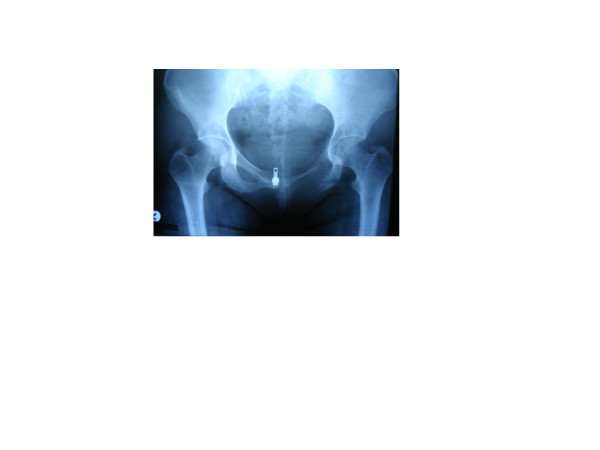
**Plain X-ray films showing isolated loss of the left inferior pubic ramus and thinning of the superior with no other bony anomalies**.

**Figure 2:**
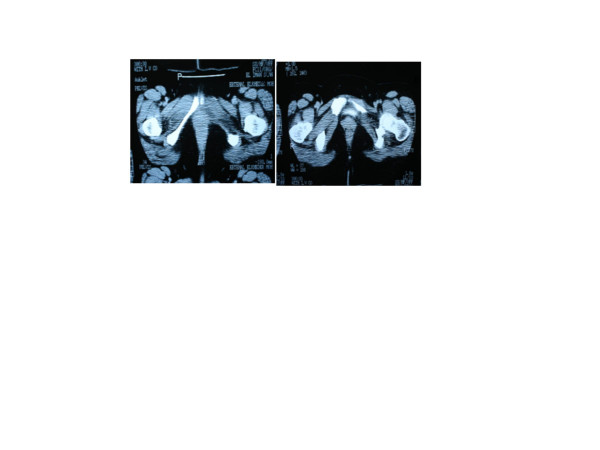
**Computed tomography scans**. (a) Isolated loss of the left inferior pubic ramus and thinning of the superior with normal muscular attachment. (b) The thin left superior ramus.

**Figure 3:**
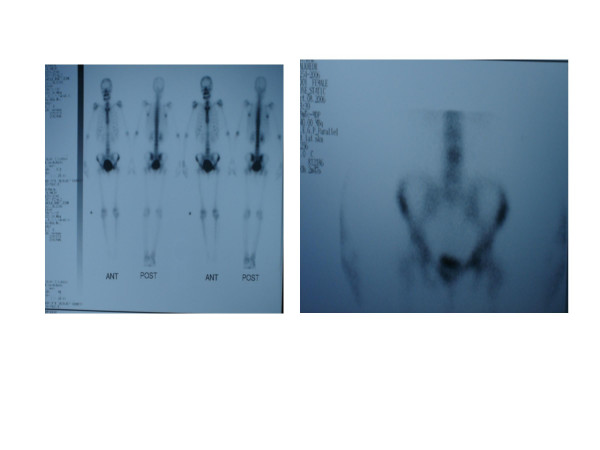
**A bone scan was performed and excluded any osteolytic lesion, bone rarefaction, cysts or neoplasm**.

Whole body imaging started with plain X-ray films which showed isolated loss of the left inferior pubic ramus and thinning of the superior, with no other bony anomalies (Figure 1). Computed tomography scans also showed the same findings with normal muscular attachment (Figure 2a) and a thin left superior ramus (Figure 2b). The same findings were obtained from magnetic resonance imaging scans. A bone scan was performed and this excluded any osteolytic lesion, bone rarefaction, cysts or neoplasm (Figure 3).

Isolated loss of the left inferior pubic ramus and thinning of the superior were detected, raising the question of whether the lesion was a secondary osteolytic lesion, a primary osteolytic lesion or due to endocrine disease.

## Discussion

Review of human limb malformation syndromes revealed a number of clinical syndromes associated with hypoplasia of ischiopubic bone: small patella syndrome, nail-patella syndrome, ischiopubic-patellar hypoplasia, and ischiopubic hypoplasia. All described pubic bone defects as well as other bony or soft tissue anomalies [5].

Small patella syndrome (SPS) is characterized by patellar aplasia or hypoplasia and by anomalies of the pelvis and feet, including disrupted ossification of the ischia and inferior pubic rami [6]. Bilateral absence of the patella in an 11-year-old girl with absence of the ischial and inferior pubic rami bilaterally, together with skeletal and soft-tissue deformities, was reported by Habboub and Thneibat and may represent a unique syndrome [7].

Hypoplasia of the ischiopubic region together with spinal dysraphism and scoliosis as well as bilateral aplasia of the patella is an extremely rare anomaly [8]. Genitopatellar syndrome is a newly described disorder characterized by absent and/or hypoplastic patellae, lower extremity contractures, urogenital anomalies, dysmorphic features, skeletal anomalies and agenesis of the corpus callosum [9]. Unilateral hip dislocation in conjunction with ipsilateral absence of the pubic bone, an undescended palpable testicle and hypospadias collectively form a syndrome that has not been reported in the orthopaedic literature previously [10].

All of these clinical syndromes show multiple bony and soft tissue anomalies [5-10], but in this case there was isolated loss of the inferior pubic ramus without any concomitant bony or soft tissue anomalies. Also, there was no association with genital anomalies. To the best of our knowledge, a case of this type has not been described previously in the literature.

The patient presented here remained free from any complaint for 35 years, a fact that reflects reported data of patients aged from 20 to 70 years, where the main complaint at consultation was with the knees due to patellar instability and pain [11]. Many patients present early in their lives, but a lack of significant clinical complaints was also reported in a 77-year-old woman with nail patella syndrome [12].

## Conclusion

Hypoplasia of the ischiopubic region is described in some syndromes together with other bony and soft tissue anomalies. However, to the best of the author's knowledge, isolated loss of the inferior pubic ramus without any concomitant bony or soft tissue anomalies has not been reported previously.

If the lesion in this case is congenital, it is unusual in being isolated with no other visceral or bony manifestations. Alternatively, the lesion may be secondary to an eroding traumatic hematoma or vascular insult. The etiology of this lesion in this patient remains unknown.

## Competing interests

The author declares that they have no competing interests.

## Consent

Written informed consent was obtained from the patient for publication of this case report and accompanying images. A copy of the written consent is available for review by the Editor-in-Chief of this journal.
